# Book Reviews

**Published:** 1801-06

**Authors:** 


### CRITICAL RETROSPECT

### OF

### MEDICAL AND PHYSICAL LITERATURE.

[ FOREIGN AND DOMESTIC. ]

*Recherches Phyfiologiques,* i. e. Phyfiological Refearches on Life and Death, &c. By XAV. BICHAT, &c. &c. Part the Second.

THE fecond part of this work is far fuperior to the firft, becaufe, inftead of chimerical fpeculation, we meet with experience and reality, afcertained by the moft ingenious experiments made on animals. It is divided into 12 articles, in which the author examines the different kinds of death, originating either in the heart, lungs, or brain; for the ceffation of life begins fometimes with the death of the heart, before the lungs or the brain ceafe to live; fometimes with the death of the brain, whofe functions difcontinue, before the heart and lungs die, &c. according to which order the author treats his fubject.

ART. 1. *General Confiderations on Death*, a recapitulation of what has been faid in the laft article of the firft part. The influence of fociety has rendered the natural death rarer; and cafual death, occafioned either by accidents or difeafes, is the particular fubject of the author's contemplation.

ART. 2. *In what manner does the death of the heart produce the death of the brain?* The inftruments by which the heart acts upon the brain, are not the nerves, but the blood-veffels, and from that fource the death of the brain is to be derived. The red or arterial blood of the heart acts upon the brain in a double way: 1. By the habitual motion which it imparts to the brain. 2. By the nature of the principles which conftitute it. Hence it appears, why a ceffation of the functions of the left ventricle of the heart brings on death death to the brain in an indirect way. A ligature of the carotids did not suddenly kill, on account of the anastomosis with the arteria vertebralis. The author endeavours to explain why animals, into whose system of vessels air is brought, suddenly die, from the cerebral motion being destroyed by the contact of the air, whence the function of the heart and the circulation are stopped. Young horses, whose blood-vessels were filled with air, tumbled down in convulsions, and expired a few minutes after.

ART. 3. *In what manner does the death of the heart produce the death of the lungs?* In the function of the lungs, two phenomena are observed; the first of which is purely mechanical, and consists in the elevation and depression, in the dilatation and contraction of the thorax, &c by the muscles and diaphragm, and in the entry and exit of the air; the second is, chemical. When the heart ceases to act with the veinous blood, the chemical process of the lungs is destroyed; but when the heart dies first with the arterial blood, the mechanical motions cease; because the death of the brain, which only acts upon voluntary muscles, occasions the cessation of the action of the parts belonging to the motive powers of the thorax.

ART. 4. *In what manner does the death of the heart produce the death of all the organs?* In *animal life*, 1. Because every organ is no more stimulated by blood; and, 2. Because the brain, by wanting itself the necessary stimulus, is unable to communicate it to the organs subordinate to it. In the *organic life*, 1. Because the necessary excitation is deficient. 2. Because the materials requisite for the continuation of this life are wanting. The author makes here some ingenious remarks on the excitation of the organs by the motion of the entering blood, and particularly on the different motions of the brain, which consist in an alternate elevatio and collapsus, that are caused by the entrance of the blood, &c.

ART. 5. *In what manner does the death of the lungs produce the death of the heart?* The suppression of the mechanical motions of the lungs is occasioned by different causes. The author, however, agrees with GOODWYN, that the folds of the larger and capillary vessels in and near the air-cells, do not obstruct the circulation of the blood, when *expiration* is for a long time continued; and he thinks likewise, that an extension of the air-cells, by a long continued inspiration, does not disturb the circulus sanguinis minor. The cessation of the mechanical motions stops the circulation, by destroying the chemical process. Mr. GOODWYN, however, is of opinion, that the cause of asphyxia may be derived from the black or veinous blood not sufficiently stimulating the left ventricle. From a want of air fit for respiration, the process of oxygenisation cannot proceed, and the body dies; because the left heart being paralysed, pushes forward neither black nor red blood. This opinion, however, appears insufficient to our author, on which account he proposes another, which likewise labours under some difficulties. Though he admits, in some measure, the paralytic effect of the black blood, yet he thinks that it still continues for some time time to ftimulate the red blood veffels almoft as much as the red blood itfelf; but as foon as it penetrates the inner texture of the heart, and the reft of the organs, it deftroys the life of every muf-cular and nervous fibre; but whether this is done by a fuperabundance of carbon or hydrogen, is not yet explained.

ART. 6. *In what manner does the death of the lungs produce the death of the brain?* The black blood, by penetrating the texture of the brain, deftroys its action. On transfufing red blood into the temporal artery, the animal remained lively and well; whereas the injection of veinous bl od killed it in a moment, and injected ink, gall, urine, ferum fanguinis, had the fame effect. When thefe fluids were injected into the arteria cruralis, they occafioned a ftupefaction and paralyfis, but never death. The blood from the arteria carotis of a dog, affected with afphyxia, when injected into the carotid of another dog, had a mortal effect.

ART. 7. *In what manner does the death of the lungs produce the death of all organs?* The author treats here of the procefs of re-fpiration and coloratio fanguinis, which is performed in the extremities of the bronchiæ. The art of changing black bloo' into red, proceeds always in proportion to the quantity of air fit for refpiration, that is contained in the lungs. An animal, breathing hydrogen and carbonic gas, becomes reftlefs after three minutes, and 5 minutes after the blood of the carotids becomes black. The black blood penetrates the mufcles, nerves, fkin, &c. hence the blue and livid appearance of the neck, cheeks, face, &c. in drowned people, or thofe that are fuffocated by the fmoke of coals. The blood of the arteria umbilicalis in a pregnant bitch, lying in afphyxia, was quite as black as that of the vena umbilicalis. There exifts no difference of colour in the blood of the fœtus of the Guiney pig. The chemical procefs in the lungs ceafes always fooner than circulation; and it is on this account that in dead bodies we always find black, and never red blood.

ART. 8. *In what manner does the death of the lungs produce the general death?* The fucceffion of the phenomena of general death, occafioned by the death of the lungs, is related here. The afphyxiæ do not merely depend on the penetration of the black blood into the texture of the lungs, brain, &c. but, 1. They arife from a want of air fit for refpiration. 2. They come on when, befides this, a deleterious fluid enters the lungs, as mephitic air, &c. which particularly feems to affect mortally the nerves of the lungs by entering into the blood, and even the brain itfelf.

ART. 9. *In what manner does the death of the brain produce the death of the lungs?* The author afferts, that the brain has no direct influence on the lungs, and death is imparted from the brain to the lungs through the medium of the diaphragm and the intercoftal mufcles.

ART. 10. *In what manner does the death of the brain produce the death of the heart?* The brain has likewife no immediate influence on the heart; but this dies after the function of the lungs being deftroyed, whence the death of the heart arifes. The author avails himfelf himfelf of the Galvanic experiments, in order to explain this difficult part of his fyftem, in which attempt he difplays much ingenuity and acutenefs.

ART. 11. *In what manner does the death of the brain produce the death of all organs?* The brain has no immediate influence on the functions of the organic life; but the death of the brain can only effect the death of the reft of the animal organs by producing the death of different intermediate organs.

*Ideas on the ganglia and the nervus fympatheticus magnus.* All organs, that receive their nerves from ganglia, are not fubordinate to the action of the brain and to the will. The fympathetic nerve confifts in a re-union of fmall nervous fyftems, each of which has a ganglion as the central point, independent of one another; they anoftomofe with the medulla fpinalis, and with each other. The nerves that arife from thefe fmall fyftems, are not to be confidered as dependent on the great fyftem of nerves which belong to animal life.

ART. 12. *In what manner does the death of the brain produce the general death?* As the death of the brain is not capable of directly deftroying the functions of the inner life, the following fucceffion in the ceffation of the general life takes place. 1. Senfibility and locomotion ceafe. 2. Diaphragm and the intercoftal mufcles become paralytic. 3. The mechanifm of refpiration is deftroyed. 4. The chemical procefs of the lungs ceafes. 5. Black blood enters into the fyftem of red blood. 6. Circulation is obftructed. 7. The heart dies, and the circulation entirely ceafes. 8. The organic life, and, 9. The animal heat, are extinguifhed. And, at laft, 10. The white organs, as they are called, die, becaufe their nourifhing fluids are almoft entirely independent of the great circulation.

---

*Ueber die verfchiedene Form*, &c. i. e. On the different Form of the Os Intermaxillare in different Animals; by GOTTHELF FISCHER, Librarian at the Univerfity of Mentz. Leipfic, 1800. pp. 151, 8vo. with 3 plates.

THIS is an excellent work on a very important fubject in Comparative Anatomy; and has additional intereft, becaufe it gave rife about two hundred years ago to fome literary debates, the decifion of which is equally interefting both for anatomy and for the natural hiftory of the human fpecies. For though GALEN had mentioned in his Treatife on Ofteology, amongft the bones of the fkull, the intermaxillar bone, VESALIUS proved, however, that this was wanting in men, and that confequently the work of Galen had not been compofed after human bones; for which affertion the Galenifts feverely expoftulated with him. Mr. FISCHER having again examined this fubject, afferts, likewife, that there is no trace of an intermaxillar bone in the human fpecies, the rimula femilunaris having only a very diftant analogy with it. He is alfo of opinion, that this bone exifts in the reft of Mammalia, and conftitutes a chief difference from man. It is true, that there are feveral animals, in which it has not yet been difcovered, as in the *Vefpertilio ferrum equinum, Sorex Arabicus*, and *Bradypus tridactylus*; but it is not not improbable, that on account of its minutenefs and flight cohefion with the fkull, it has hitherto efcaped obfervation.—The author determines the form and fituation of this bone in general, and its general differences, the proportion it keeps to the foreteeth, &c. He relates farther the fpecific differences in many animals, the fkulls of which he had an opportunity of examining in the National Mufeum of Paris, &c. Mr. F. attributes likewife an intermaxillar bone to fome birds, fifhes, &c. The plates reprefent the intermaxillar bones of different exotic animals, fifhes, &c. of which thofe of the *Chætodon roftratum* and Trigla volitans are particularly remarkable.

---

### *Critical Survey of the lateft Theories of difficult Dentition.*

[ Concluded from No. XIX. pp. 269, 271. ]

*In medio Veritas!*

After having ftated the theory of Dr. WICHMANN on difficult dentition, by which he attempts to overthrow entirely the exiftence of it, as a morbid phenomenon, we think it incumbent upon us to propofe and difcufs what might and has been faid againft this opinion, fo diametrically oppofite to common obfervation and experience. On this account we doubt not, but that by far the greateft part of our readers will have been furprifed at the paradoxical affertions of Dr. WICHMANN, and recapitulated in their minds the different contrary arguments, that either their own experience, or the obfervations and authority of medical writers, may fuggeft to them. It can be eafily conceived, that in Germany alfo many antagonifts have arofe againft the above theory, equally powerful in their arguments and authority as practitioners. However, though we are fully convinced, that many points in the opinion of Dr. WICHMANN, cannot be well admitted, as being contrary to impartial obfervation, yet we ought not to deny, that this great practitioner has occafioned by his ideas, medical men to fubmit fuch an important fubject to new examination, and to throw more light upon it, by drawing from experience practical refults; in fhort, to fix more accurately the limits of the caufes of infantile difeafes than hitherto has been done.

It is certain, that hydrocephalus, fcrophula, acor primarum viarum, &c. have been frequently overlooked; and the fymptoms attending thefe morbid affections, falfely afcribed to a difficult dentition, particularly as they in a great meafure occur at the period of teething. However, on the other fide, by adopting the ideas of Dr. WICHMANN, practitioners are unavoidably led to derive all morbid affections of the infantile age from an acor in the ftomach, hydrocephalus, fcrophula, &c. and by difregarding entirely dentition, to incur the danger of not employing that mode of practice, which is properly indicated.

On the perufal of Dr. WICHMANN's opinion, the chief point which he has premifed to his other arguments is, undoubtedly, that the the gums, or thofe parts, which by the irritation of a tooth breaking through, may excite pain, and by confenfus, the reft of fymptoms thought to attend difficult dentition, are by no means endued with fuch a degree of fenfibility, capable of producing thofe affections; but that the gums, which, at the fuppofed want of a perioftium, are the only parts to be irritated, are nearly deprived of all fenfation, being a fpongy, fenfelefs fubftance. This affertion ought, therefore, to be firft confidered; and it deferves the firft place among the arguments that have been brought forward againft the above opinion.

1. *That the gums are really a very fenfible and irritable part.*—— No part of the body into which nerves do penetrate, can be faid to want fenfibility; and as the gums are undoubtedly provided with feveral nervous fibres, it ought to poffefs the perception of pain. Of the prefence of nerves in that part, no particular mention is made by the author of the above theory; on the contrary, he feems to treat the expreffion of SAUVAGES, "*a compreffione nervi,*" with ridicule, and to pay to the nerves not that attention which they deferve. Every body, however, may perfuade himfelf of the exiftence of nerves in the gums, by confulting the works of HALLER, SOEMMERING, MONRO, and particularly MECKEL, (*de quinto pare nervorum cerebri,* p. 63 and 96). The nerves that go to both thefe parts, originate from the fifth pair of the cerebral nerves, and are the following.—The ramus pofterior, or minor of the nervus palatinus, fends two branches, one on each fide, to the molar teeth of the maxilla fuperior, which again dividing themfelves in two branches, extend to the foremoft teeth, and emit fibres to the palate, and particularly to the gums. The ramus tertius of the palatinus, or of the alveolaris maxillæ fuperioris, feparates in the dentatem pofteriorem, emitting branches to the molar teeth, antrum Highmori and Orbita, and in the dentatem anteriorem maxillæ fuperioris, which provides the foreteeth, particularly the eyeteeth. The upper lip receives likewife nerves from thefe branches, which go to the orbita, and it is highly probable that they extend to the gums, though, on account of their fubtility, they cannot be purfued by the knife. The third chief branch of the par quintum, or the nervus maxillaris inferior, proceeds, after having difpatched a few branches to the mufcles of the os hyoides, to the canal of the maxilla inferior, and provides every alveolus with a nerve, and gives befides to every molar tooth a fmall additional branch. All the nerves of the fifth pair, and the portio dura of the 7th and the 9th pair, are combined by an anaftomofis with the maxillaris inferior, whofe third branch is diftributed to the falival glands, mufcles of the tongue, and to the gums.

2. *The hiftory of the formation of the teeth, seems to prove the exiftence of difficult dentition.* The ftamina of the teeth, furrounded by a vafcular membrane, are fituated in the alveoli, which, according to anatomifts, have no opening before the teeth break through, fome but clofed with a long fubftance, and covered by the gums. At the advancing formation of the tooth, this is to make its way through through the bony cover by its root that, while increafing, is fupported by the bafis of the alveolus preffing the head of the tooth againft the cover of the alveolus, till yielding, it allows the tooth to pafs by degrees. This is what they call the fhooting of the teeth. The ftronger and harder the cover is, and the more refiftance it fhows, the more the tooth preffes the bottom of the alveolus, by which the nervous branches that go to the root are irritated, which particularly happens in the eye-teeth. From a preffure of thefe nerves, which have a very extenfive fympathy, fuch vehement fymptoms may arife as are recorded in the works of Bartholin, Tiffot, &c. The tooth after having perforated the bony cover muft pierce thro' a proper vafcular membrane or perioftium, as we fee from the works of SOEMMERING, who fays, "that the head of the tooth having penetrated the perioftium appears bare, as the membrane only incloses thofe parts of the tooth that are covered by the gums, and lie in the alveolus (*On the ftructure of the human body, T.* 1. p. 207, in German). L. HEISTER calls this membrane, "Membranam vafculofo nerveam." The gums, through which the tooth is then to pafs, are in all points connected to the perioftium of the jaws, which naturally increafes the fympathy.

3. If we even fuppofe with Dr. WICHMANN, that the gums are quite fenfelefs, or at leaft provided with no particular degree of fenfibility, that no perioftium exifts, there is ftill no reafon to affert, that thofe parts are equally deprived of fenfibility during dentition. For it is a known fact, that parts poffeffed of little or no fenfibility, as the bones and cartilages, receive a great perception of pain, or become painful in a morbid ftate, a circumftance which ought to be particularly regarded in this queftion.

4. *The ftate of the gums in teething children is frequently fuch as denotes the exiftence of an inflammation.* It is very obvious to obfervation, that the gums are in many cafes extremely painful, and that children hardly bear the flighteft touch without experiencing the moft painful fenfation. The inner parts of the mouth, and efpecially the gums, feel very hot, and caufe a burning fenfation in the finger. It has befides been mentioned, that, when the teeth have broke through, great pain is occafioned by the tenfion of two or three flefhy fibres happening to remain on the tooth, which being cut through, the pain immediately ceafed. We find, however, that topical pains are denied by Dr. WICHMANN to take place, though he admits at the fame time, even in an eafy dentition, a tickling and prurient fenfation, which indeed differs only in degree from what we call pain, into which it may of courfe be changed under certain circumftances. There is undoubtedly a topical irritation in every dentition, which is proved by feveral other phenomena, as falivation, a continual effort of children to relieve themfelves in a lefs degree of irritation by biting fome hard fubftances, the greater force which they then apply in fucking, and other fymptoms that are commonly obferved. Dr. WICHMANN thinks thofe cafes rather anomalous, in which the inflammation even extends itfelf to the neighbouring parts; however, it ought to have proved to him, how liable thofe parts are to pafs over from a flight degree of irritation to inflammation. It is likewife not uncommon, that adults feel the moſt painful ſenſation at the appearance of the laſt tooth, dens ſapientiæ, and cannot ſuffer then the ſlighteſt touch on the upper part of the gums; hence it is probable, that in ſuch a tender age, when dentition takes place, ſimilar pains may be produced. The redneſs of the gums moſt frequently attends a difficult dentition, and though it does not always occur, it ſeems to prove, that the gums become inflamed by the irritation of the teeth cutting through, of which it is an additional ſymptom.

5. Dentition proceeds in two periods; in the firſt of which, that is properly called the ſhooting of the teeth, the tooth irritates the membrane in which it is incloſed, the ſides of the alveoli and the parts adjacent. No ſymptom of inflammation or ſwelling of the gums is perceived: In the ſecond period the tooth breaks through, ſometimes on a ſudden, and without any pain. In ſome inſtances, an interval takes place between theſe two periods, but frequently the formation of the tooth proceeds uninterrupted, till it appears in its natural ſize. According to this, two ſpecies of difficult dentition may be likewiſe admitted, the true and the ſpurious, the firſt of which originates in the perforation of the alveolus and in the irritation and preſſure of the neighbouring parts, the perioſtium, and the dental nerve, whence different nervous affections may ariſe without any ſwelling or redneſs being perceptible; in the ſecond ſpecies, which is more common, the gums are extended, ſwollen, and inflamed; and all the ſymptoms increaſe and become particularly urgent, when at the ſecond period of dentition, the preſſure of the nerve continues, or when the parts are irritated in this double way. Hence it may be explained, why the ſymptoms of dentitio difficilis were obſerved, without much ſwelling or inflammation of the gums, and why they often ceaſed when a ſwelling and redneſs of the gums were perceived, becauſe at the advancing growth of the tooth, the nerve and perioſtium were no more irritated, and the irritation of the gums alone not ſufficient to produce the ſymptoms that were perceived before this period.

6. With reſpect to ſalivation, a ſymptom that is often remarked at the period of teething, Dr. Wichmann is more inclined to aſcribe it to aphthæ, and to a mere irritation of the ſalival glands, than to attribute it to dentition. We may however obſerve, that aphthæ are not always concomitant with this ſalivation, which, as it keeps pace with the act of teething, may be more juſtly thought to depend on the irritation of the teeth breaking through, that acts per conſenſum on the ſalival ſyſtem. This cauſe is at the ſame time ſo near at hand, that there is no reaſon to ſearch for another we know not.

Such are the arguments that may ſerve to prove the exiſtence of an irritation in the mouth at the period of dentition, ſufficient to produce topical ſyſtems of difficult teething. It remains now to be examined, whether the general ſymptoms that have been derived from difficult dentition are really owing to idiopathic affections, as is aſſerted by Dr. Wichmann. However, before we recur to that queſtion, we beg leave to obſerve, that none of theſe general ſymptoms toms ought to be feparately regarded; but that in order to be fully convinced whether any of them arife from the above caufe, it will be the fureft way to enquire into the other fymptoms that attend dentition, and from the coexiftence and coincidence of other fymptoms, to conclude that they are caufed by the irritation of the the teeth. We readily agree with Dr. Wichmann, that fome fymptoms are owing to different caufes, not depending on dentition, or may at leaft have been produced by them, and we are not willing to deprive this gentleman of the praife he merits, for having pointed out the true fource of feveral fymptoms, otherwife attributed to difficult dentition; but we cannot forbear to confefs, that by utterly denying their origin from dentition, he has equally departed from truth. For, 1. Moft of the general fymptoms attending difficult dentition are phenomena of an affection of the nervous fyftem, and fymptoms of irritation and pain. How they are produced, appears from what we have above ftated. Convulfions are accordingly excited by the re-action of the topical affection on the whole fyftem of nerves, by which it fuffers more or lefs, according to conftitutional difference. It is, farther, not at all inconfiftent with analogy and the laws of animal oeconomy, to derive fome other fymptoms which Dr. Wichmann finds particularly abfurd to afcribe to dentition, from a confenfus of the topical irritation, viz. diarrhœa, ftrangury, &c. as we know, for inftance, that the affection of the olfactory nerve by the fmell of a medicine, that acts as a cathartic, produces the fame effect in fome perfons as if they had fwallowed it. A fever is likewife obferved to attend fome cafes of dentition, owing alfo its origin to irritation.

2. It is remarkable that a very intimate connexion and concurrence of the period of teething, and the formation of the teeth, and of thofe general fymptoms exift, which admit of no better explanation than to think them mutually depending on each other. The topical figns of dentition appear, and at the fame time the general fymptoms of it, keeping nearly pace with one another, as the laft moftly difcontinue as foon as the act of teething is performed. The obfervations and experience of the moft refpectable practitioners bear witnefs to this fact, which feems to have been not fo much attended to as it deferves.

The incifion is utterly rejected in this theory as an ufelefs operation, for which affertion he appeals to fome cafes where it was undertaken without fuccefs, and to thofe practitioners who profeffed themfelves antagonifts to that operation. The fuccefs of it, however, depends on the time and the period of dentition when it is reforted to. According to our former ftatement of the two periods of dentition, it muft naturally prove without avail when the tooth has not yet pierced through the alveolus, and fervice can accordingly be expected when the tooth begins to extend the gums; in which cafe it has been found extremely ufeful. To the authority of thofe phyficians who reject it, we can oppofe that of others who approved it, equalling thofe as well in number as in practical excellency.

excellency. Drake, Vesalius, Ettmuller, Underwood, Rosenstein, Ludwig, Fr. Hoffmann, Frank, Darwin, &c. think it an operation not only ufeful, but fometimes neceffary, which they have performed with the beft fuccefs.

Againft the general reflections which Dr. Wichmann has fubjoined, may be obferved, that nature feems not to be, in any formation of parts, fo precipitate as in the act of teething; and by her proceeding in the growth of other bones more gently, thofe affections are avoided which we remark in dentition, even when the nature and fituation of thefe parts did admit of a fimilar irritation of the fenfible and irritable fibre. It is by no means to be fuppofed, that dentition fhould be always attended with fymptoms that render it difficult; but feveral circumftances muft concur, in order to produce the above morbid ftate; and as at the period of approaching puberty and fluxus menfium the conftitution may be affected by the change that is then experienced, this may likewife take place in that operation of nature. How eafily the animal body is influenced by the act of teething, appears from the obfervations of naturalifts, who have remarked that the brute creation fuffer greatly by dentition; and it is related that many young animals, horfes, lions, &c. fall a facrifice to difficult dentition. When we do not perceive thofe fymptoms in adults, fuffering the fevereft tooth-ach, we fhould always recollect, that dentition of children proceeds in a tender age, where the irritability of the body is very great, and the confenfus more extended than afterward. This may fuffice to fhew, that dentition ought not to be exploded from the lift of morbific caufes, as Dr. Wichmann intended by his obfervations.

---

### *Mr. Whately, on Strictures in the Urethra.*

(Concluded from p. 497—482 of our laft.)

Mr. W. commences his own *method of treating Strictures in the Urethra, by the Cauftic,* with the following obfervations and cautions, viz.

"Before entering on the particulars of my propofed method, it may not be improper to repeat, that it is effentially neceffary, in our prefent imperfect acquaintance with the cauftic, to endeavour to dilate all ftrictures of the urethra by means of common bougies, before any attempt be made with the cauftic to effect their cure. Mr. Home, indeed, informs us, that this has been done in almoft every inftance adduced in favour of the cauftic. As, however, even in the cafes related by him, there are fo many inftances of ferious effects produced by the cauftic, it is highly neceffary to enforce this rule; a rule which, I fear, is not very ftrictly obferved by fome practitioners.

"According to our prefent experience, I would confine the practice of applying this remedy altogether to fuch ftrictures of the urethra, as are either utterly impervious, or fo contracted, as to be incapable of dilation by the common bougie: for the urethra, even in its healthy ftate, being tender and irritable, and connected connected with parts of great importance in the fyftem, all violent remedies fhould be very cautioufly applied to it.

"One of the objections made to the ufe of cauftics, for removing ftrictures of the urethra, is the impracticability of confining their action to the conftricted part. But, I muft confefs, I do not perceive the force of this objection; for I think, that they may be almoft always applied to fuch part with great nicety. Wifeman, and other ancient furgeons, applied efcharotics of different kinds, by means of adhefive plaifter at the end of bougies, in order to remove obftructions and excrefcences, which they fuppofed to exift in the urethra; the former likewife ufed a canula for the purpofe of introducing a cauftic. This practice, however, in confequence of the danger found to arife from it, was laid afide for near a hundred years; but was revived by Mr. Hunter, who, as remarked at the beginning of the former effay, introduced the lunar cauftic to ftrictures of a certain defcription, at firft through a canula, and afterwards by enclofing it in the end of a common bougie. Very lately, Dr. Sherwin has ingenioufly propofed to remedy the inconveniences of the cauftic bougie, by applying a certain quantity of the lunar cauftic in powder, at the end of a warm whalebone bougie, to which it is to be fixed by adhefive plaifter, or any other vifcous fubftance. This method, however, does not appear fufficiently to fecure the fides of the urethra from being injured by the cauftic in its paffage to the ftricture; befides, there is danger of the plaifter being detached from the whalebone, before it has reached the ftrictured part.

"About two months before I heard of Dr. Sherwin's propofal, I made feveral effays to difcover a fubftance that would make the cauftic adhere firmly to the bougie, and yet not hinder its diffolving on the conftricted part. The following method will completely anfwer both thefe purpofes: Touch an eighth, or from that to a quarter of an inch, of the end of a bougie of any fize, with a fmall brufh dipped in common glue, as ufed by mechanics; and let the coating of glue be as *thin as poffible.* The glued end muft be immediately applied to a given quantity of powdered lunar cauftic, put upon a piece of writing paper; this fhould be done, by alternately putting its different fides to the cauftic, until the whole of it adheres. The bougie in this ftate muft be laid in fome dry place to harden, which effect will take place in a few hours. When it is fufficiently hardened, the glued end muft be gently rolled to and fro upon a table, with a bit of fmooth wood, about four inches fquare, till it is perfectly level and fmooth. If the bougie be very hard, or the weather cold, this end fhould be previoufly warmed a little by a fire. The part thus covered with cauftic fhould then be very lightly rubbed with a bit of bees wax, with the intention of giving it a very thin coating of this fubftance. After this, let it be kept for ufe in a *glafs veffel well clofed.* The glue fhould be made of fuch a degree of confiftence only, as will make the cauftic adhere to the bougie, and become hard when expofed for a moderate time to a dry air. If it be made too ftiff, it will be attended attended with the inconvenience of requiring a longer time in being diffolved, when applied to the conftricted part; on this account too, the coating of wax ought to be as thin as poffible.

"The advantages attending the cauftic bougie prepared in this manner are obvious.

"In the firft place, the bougie may be of any fize; even the fmalleft fize can, by this method, become the vehicle of this powerful remedy; and may be readily paffed into, or a little beyond, fuch ftrictures as are extremely narrow; or fuch as are attended with a confiderable contraction of the orifice of the urethra.

"Secondly. From the protection afforded by the wax coating, no part whatever of the cauftic touches the fides of the urethra in its paffage to the ftricture.

"Thirdly. A determinate quantity of the cauftic may be applied with certainty.

"Fourthly The cauftic cannot be feparated from the bougie.

"Fifthly. The cauftic may be made to act on the whole furface of the ftricture at each application.

"Sixthly. Where there are more ftrictures than one, and it is thought advisable, to attend to one only at the firft; the cauftic may be directed, and confined in its action, to any particular ftricture, upon which the practitioner may wifh it to operate, in preference to the reft.

"Seventhly. Fixing the cauftic with glue has this additional recommendation; we can attach it with perfect fafety to the extremity of a bougie, and thereby apply it with more certainty to an impervious ftricture, than is practicable with the common armed bougie.

"When we have determined to ufe the cauftic bougie thus prepared, the diftance of the conftricted part from the extremity of the penis fhould be accurately meafured, in order to apply it with certainty to this part only. This may be done with a common bougie. The exact fize of the canal at the part fhould likewife be afcertained by the fame inftrument. This in general may be readily done. They who are ufed to pafs a bougie of a proper fize through a ftricture, can always tell when the point of this inftrument is about to enter it. When it firft touches the ftricture, it generally ftops; but on preffing the bougie gently on, it evidently feels as if its point entered a paffage, which embraces it on every fide. The bougie paffes on afterwards more or lefs freely, according to the fize of its upper part, and the opennefs of the paffage beyond the ftricture.

"Having afcertained thefe points, it will be proper to choose a bougie armed with cauftic, in the manner already mentioned, of a fize rather lefs than the conftricted part. A piece of fine white thread fhould then be tied round the bougie, at the exact diftance of the ftricture from the end of the penis, and another thread a quarter of an inch nearer the external end. By the firft of thefe marks it will be known with great accuracy, when the bougie enters the conftricted part; and the fecond will determine how far it is pufhed beyond beyond it. This armed bougie (previoufly oiled) may now be paffed down to the ftricture, and about the eighth of an inch into it, and continued in this fituation from five to ten minutes, now and then alternately moving it forward and backward, about the eighth of an inch each way, in order to wipe off any mucus which may have collected upon the cauftic, and to affift in wiping off the cauftic itfelf. On withdrawing the bougie, all the cauftic will be found to be diffolved; whence we may fairly conclude, that it has been expended entirely on the conftricted part.

"If the ftricture be open enough to admit a bougie of a moderate fize, fuch a bougie, armed with cauftic, may very readily be paffed into it, or a little beyond it; but, if the ftricture admit only a very fine bougie, and cannot be dilated fo as to receive one of a larger fize, great care fhould be ufed in paffing a cauftic bougie of a fmall fize into the ftricture. For if this fhould not be effected, and its extremity be bent in endeavouring to pufh it forward, much effect cannot be expected from the cauftic, as it will be diffolved at a part of the canal anterior to the ftricture. From what has been faid, it is evident, that the fuccefs of applying the cauftic by this method will depend very much upon the nice manner in which it is performed.

"With refpect to the quantity of cauftic which ought to be applied to a ftricture, I am convinced, that, on the firft trial, it fhould in no cafe exceed one-twelfth part of a grain."

As Mr. Cartwright has propofed a new method of applying the cauftic in thefe cafes, and difapproves of that recommended above, (See page 513) we fhall take this occafion to fuggeft a few obfervations on his plan.

The method which Mr. C. adopts of applying lunar cauftic in powder to a ftricture of the urethra, by means of the broader end of a common bougie introduced through a flexible gum canula, appears to us to be more uncertain in its effect than that which Mr. Hunter recommends of applying a bit of the fame cauftic in a port crayon through a flexible filver canula, or by means of his armed bougie now in ufe.

In the firft place, as no means are employed to make the cauftic adhere to the end of the bougie, the quantity of it actually applied to the ftricture muft be uncertain.

Secondly, The *flat* end of a bougie *cannot* be applied with certainty even to the anterior part of a ftricture when it is much contracted, whereas the conical point of the cauftic in the port crayon is well adapted to this purpofe.

Thirdly, As it is not propofed by this method, that the cauftic bougie fhould be paffed into, or a little beyond the ftricture, even in thofe cafes which will admit a bougie of a middle fize, much of the benefit to be expected from the ufe of the cauftic will be loft in applying it to the anterior part of the ftricture only, as the liquefied cauftic will flow anteriorly; for all fluids in the urethra flow towards the extremity of the penis by means of the mufcular action of its canal.

Fourthly,

Fourthly, The *flat* end of a bougie thus armed with cauftic, *cannot*, from its fize, be paffed into or through a very narrow ftricture; and as the cauftic is of more ufe in ftrictures of this kind than in any others, it muft be confined in its application to the anterior part of them only, which, as already obferved, is an uncertain mode of applying it.

The objections which Mr. C. has made in his Poftfcript to Mr. W's mode of applying the cauftic in thefe cafes, do not appear to us well founded.

When the whole of the cauftic fixed upon the end of a bougie by means of glue, is perfectly diffolved and liquified upon a ftrictured part of the urethra, it muft of courfe immediately act upon that part, and if the bougie be fuffered to remain upon the ftricture until this effect takes place, (which, according to Mr. Whatley) requires to be done from 5 to 10 minutes) is it poffible that the withdrawing a bougie thus *perfectly freed* from its cauftic, along the found part of the urethra, can be an *infuperable objection* to its ufe, and attended with *mischievous confequences?*

That the very extremity of a bougie, armed with cauftic by means of glue, muft be immerfed in the moifture of the urethra, which diffolves the cauftic, is certainly true; but it is evident, that the very fmall portion of liquid which adheres to it, muft be wiped off the moment the bougie is attempted to be withdrawn. Befides, were this an *infuperable objection* to this mode of applying the cauftic, it would be equally an *infuperable objection* to the mode of applying it by the flat end of a bougie, as recommended by Mr. C. As fuch an end would retain a portion of the liquified cauftic upon its furface, more of it would alfo unavoidably adhere to the extremity of the canula; and when both were withdrawn, *mifchievous confequences* muft neceffarily arife to the found part of the urethra, if fuch confequences enfue upon withdrawing the bougie after Mr. W's method of applying the cauftic.

This powerful remedy may, indeed, be in a very trifling degree weakened by its admixture with the glue; but this appears to us to be even an advantage in fome cafes, as lefs mifchief or pain may thereby enfue from its ufe in very irritable habits; and the activity of this application is fuch, (if it be kept in a glafs veffel well clofed, as directed by Mr. Whately) that the very fmall quantity of thin glue with which it is mixed, cannot, one would fuppofe, prevent its acting with power on any furface to which it is applied, which appears to be proved by the cafes Mr. Whately has given; and whenever a greater effect is neceffary, it would appear to be a very eafy matter to make a little addition to the quantity, after it has been proved that the lefs quantities have not had the required effect.

As Mr. C's object is the improvement of his profeffion, he will not be difpleafed at thefe hints, which we hope he will improve.

---

Dr.

### *Dr. Baeta's Comparative View, &c.*

( Concluded from p. 388—391. )

"When the torpor from exhauſtion of ſenſorial power affects other parts of the ſyſtem, which have their actions aſſociated with thoſe of the ſtomach, as, for inſtance, the ſpleen, liver, &c. the ſtomach falls into a torpor, from defect of excitement of the ſenſorial power of aſſociation, and ſo the arterial ſyſtem, till a general torpor is formed, which conſtitutes the cold fit, (Sect. xvii). Now during the cold fit, an accumulation of ſenſorioal power of aſſociation takes place in the ſtomach, arterial ſyſtem, &c. which overbalances this defect of excitement of the ſenſorial power of aſſociation: conſequently, theſe parts are thrown into increaſed actions. This conſtitutes the hot fit, which, according to the accumulation of the ſenſorial power of aſſociation, and to the ſtimuli applied to it, will produce various effects (Princip. 4. and note *(n)* Princip. 15). Thus either theſe increaſed actions may be proper, to reduce the ſenſorial power of aſſociation, accumulated during the cold fit, to its juſt limits, and at the ſame time to affect, by means of aſſociate motions, (Princip. 5. and 8.) that part which is torpid from exhauſtion of ſenſorial power, ſo as to reſtore its juſt degree of deriving ſenſorial power from the brain and ſpinal marrow; and then the fever is cured: or theſe increaſed actions merely reduce the ſenſorial power of aſſociation to its natural ſtandard, while the ſpleen, liver, &c. remain yet in a torpid ſtate, which, either by its degree, or by the concurrence of other cauſes, may induce again the torpor of the ſtomach, &c. in conſequence of defect of excitement of the ſenſorial power of aſſociation. Hence various kinds of intermittent fevers, or theſe increaſed actions, may be in ſuch a degree, as to occaſion ſenſation. Hence inflammatory fevers (Princip. 15). Or laſtly, theſe increaſed actions may, in conſequence of their violence, produce a ſmaller, or greater, or complete exhauſtion of ſenſorial power, in ſome part eſſential to life. Hence various kinds of continued fever with arterial debility (Sect. xx, xxi, and xxii,) or even death (Princip. 15).

"From Sect. xvi.—xxiii. it will appear, that Dr. Darwin's Doctrine of Fever explains the various phenomena which take place in this diſeaſe: viz. it accounts 1ſt, for the formation of the cold fit, and decreaſed actions of the ſyſtem, which are obſervable in it (Sect. xvii.): 2dly, for the formation of the hot fit, &c. (Sect. xvii.): 3dly, for the phenomena which attend the hot fit of fevers with arterial ſtrength (Sect. xix. and xxiii): 4thly, for the intermiſſion of fevers (Sect. xxiii.): 5thly, for the change from intermittent to continued fevers (Sect. xxii.): 6thly, for the phenomena which take place in ſome continued fevers with arterial debility; that is, the decreaſed actions of the ſtomach and arterial ſyſtem, as evinced by the want of appetite and weak pulſe; and for the increaſed actions of the capillaries, as evinced by the increafed heat (Sect. xx.): 7thly, for the abfence of the increafed actions of the capillaries, in fome fevers with arterial debility, as evinced by the abfence of increafed heat over the body (Sect. xxi.): Laftly, for the long duration of continued fever with arterial debility (Sect. xxviii.). See the foregoing cafes.

"From the fame doctrine of fever the moft proper indications of cure are deduced.—1ft, According to it, we muft excite the fyftem in the cold fit of fevers, taking care, however, to proportion the ftimuli to the fenforial power already accumulated. By thefe means we prevent the accumulation of fenforial power, which may give rife either to the hot fit (Sect. xvii.), or to inflammation, &c. (Sect. xxiii.) Hence Dr. Darwin's expreffion (Zoonomia, vol. i. Sect. 12.) "The true means of curing fever "(with ftrong pulfe) muft be fuch as decreafe the action of the "fyftem in the hot fit, and increafe it in the cold fit."

"2dly. According to the fame doctrine, during the hot fit of fevers with arterial ftrength, we are led to diminifh the increafed action of the fyftem; fince by this means we prevent the inflammation, which may arife during the hot fit in confequence of thefe increafed actions, and various other diforders (Sect. xxiii.). Hence Dr. Darwin fays (Zoonomia, vol. ii. Supl. 1, 16, 9.). "The cure "of fever with ftrong pulfe (in the hot fit) confifts in the re"peated ufe of venefection, gentle cathartics, diluents, &c."

"3dly. According to the fame theory, it appears, that the cure of fevers with arterial debility and increafed actions of the capillaries (Sect. xx.) confifts in reftoring the energy of the ftomach and arterial fyftem, and in decreafing the morbid increafed actions of the capillaries. The firft of thefe is obtained, by exciting into action the torpid ftomach (and confequently the arterial fyftem) either directly as by wine, opium, bark, &c. and food in fmall repeated quantities; by flight electric fhocks paffed through it; by fomentations with water, heated to 96 or a 100 degrees of Fahrenheit's thermometer; by exciting its power of affociation with other parts of the fyftem, as by a blifter, or indirectly, as by the exhibition of emetics, or iced water, &c. Hence the remarkably good effects of wine, bark, emetics, &c. in the foregoing cafes of fever, chiefly in that of R. Feemifton, under Dr. Hope's care. The fecond of thefe indications is obtained by free admiffion of cold air, and chiefly by the ablution with, or affufion of cold water over the furface. Hence the manifeft utility of the affufion with cold water, ordered by Dr. Gregory in the foregoing cafes, and likewife of the affufion of cold water, fo much recommended by Dr. Currie. When thefe two means, viz. the invigorating the actions of the ftomach, &c. by fmall repeated dofes of ftimuli, and the weakening the energetic actions of the capillaries of the fkin, by ablution with or affufion of cold water, are ufed conjointly, they are found to be of the greateft utility in the cure of fevers of this kind.

"Laftly, from Dr. Darwin's doctrine, it appears, that in fevers, with arterial debility and decreafed actions of the capillaries (Sect.

xxi.)

xxi.) the cure confifts in reftoring the energy of the fyftem, but particularly of the fecerning veffels of the brain. Hence all thofe fubftances which may have the power of exciting thefe veffels, will be ufeful in this kind of fevers. Opium and wine are fuppofed by Dr. Darwin to poffefs this power (Zoonomia, vol. ii. Supl. 1, 16, 9). In this kind of fever Dr. Darwin recommends alfo hot fomentations to the head, fmall electric fhocks paffed through it, and fmall blifters. Might not the warm bath be ufed with utility in thefe fevers? The refpiration of oxygen gas, diluted with atmofpheric air (fays Dr. Darwin), would be ufeful in fuch fevers (Zoonomia, vol. ii. Supl. 1, 11, 7.). Might not this remedy, when well managed, be found ufeful in thofe fevers which may arife from the exhauftion of the fenforial power of irritation in the arterial fyftem? (See Sect. xxii.). The utility of infpiring oxygen gas, diluted with atmofpheric air, in fevers with arterial debility, is related by Dr. Thornton (fee Dr. Beddoes's Confiderations on the Medical Powers of factitious airs, part iv. and v. p. 135.).

"From the explanation given, Sect. ii.—vii. it is, I think, evident;—1ft. That by means of Dr. Cullen's doctrine of fever we cannot account for the phenomena which take place in this difeafe. —2dly. That from this doctrine we cannot draw any proper indication of cure in it. Likewife from what is contained in Sect ix.—xv. it will appear,—1ft. That Dr. Brown's Theory of Fever does not account either for certain phenomena, which are obfervable in the hot fit of intermittent fevers, or for the increafed heat over the body in fome continued fevers with arterial debility—2dly. That from this doctrine we cannot draw what may be called a complete indication of cure in fevers. For it always rejects a remedy, which is fometimes ufeful; and uniformly recommends one, which is at times, or generally, noxious in the hot fit of intermittent fevers:—And laftly, it always rejects one of the moft ufeful remedies in the cure of fome continued fevers with arterial debility; and alfo of intermittent fevers, when ufed during the hot fit, when the heat of the body is above the natural ftandard, &c. But from the account given Sect. xvi.—xxiii. as mentioned Sect. xxiv.—xxviii. it is manifeft, that Dr. Darwin's Theory of Fever—1ft. Accounts for the various phenomena obfervable in this difeafe—2dly. Affords the moft proper indications of cure in it; and at the fame time explains the operation of the remedies, by which thefe indications are fulfilled.

---

### *Mr. Charles Bell's System of Dissections.*

[ Continued from Numb. XXVI. p. 376. ]

We are glad of an opportunity of giving a few more obfervations on, and extracts from, this inftructive and ufeful guide to the ftudent of Anatomy and Surgery; being well convinced that one or two courfes of diffection, profecuted with the care and patience requifite for the tafk, would not only give the ftudent confiderable practical anatomical knowledge, but make him alfo a competent judge judge of the various difeafed appearances he will frequently meet with on opening dead bodies.

After a nice defcription of the mode of diffecting the abdominal mufcles, the *linea alba*, the *linea femilunaris*, &c. the Author gives the following judicious directions on Hernia, Dropfy, and Afcites, difeafes as connected with the anatomy of thefe mufcles.

"It is wrong to cut acrofs the belly in opening collections of matter amongft thefe mufcles, unlefs they have been deftroyed by the matter; becaufe the fibres of the mufcles are then cut acrofs, hence they retract, and form a gap; and at the fame time the poffibility is increafed of wounding the epigaftric artery which runs up the belly. By opening thefe abfceffes with an incifion parallel to the fibres of the mufcles, the parts are divided, without allowing the mufcles to retract; and the chance of wounding the arteries is leffened. In tapping for the dropfy, it is faid that the epigaftric artery (the courfe of which I have marked in the plate with a dotted line), is fometimes wounded, or its accompanying vein. But it fhould be expected, when thefe were wounded, that while the canula remained in the wound, diftending the orifice, they fhould not bleed. If they fhould bleed, however, they may probably be ftopped by preffing the canula obliquely to one fide. I have never feen an accident of this kind; but fuch cafes have been defcribed to me, where the deluge of waters was coagulated in the tub. Perhaps an enlarged fpleen, or fome of the vifcera touched with the trochar, may fometimes account for fuch a bleeding."

The Author's directions for the mode of opening the abdomen, to inveftigate the feat of difeafe, are clear and fimple. Thefe are the ftages: "Make a crucial incifion, at once laying open the vifcera—or, if in a female, make your incifion fo as to leave a triangular flap to fall over the parts of generation, by continuing your longitudinal cut no further than the umbilicus, and from that point, making an oblique incifion on each fide, towards the projecting point of the ilium, forming thus three triangular flaps. Then obferve whether the parts are in their natural fituation: examine the omentum, the ftomach, the fpleen, the inteftines, and then the liver and gall ducts. Then feparating the ftomach and colon, connected by the omentum, raife the ftomach, and examine the pancreas—Cutting up the adipofe membrane, examine the kidneys, by making a fection of them. Then following the ureters, examine the contents of the pelvis, &c. In making up your cafe, firft mark the general deviations from the healthy appearance of the vifcera; then detail the hiftory of the difeafe, and take notice of any anomalous appearances which feem unconnected with the principal difeafe.

"On this fubject (Difeafe of the Abdominal Vifcera) it is of importance to ftudy the nature of inflammation, of adhefions, and fuppuration, and the almoft uniform confequence of difeafe upon the peritoneum. It will be eafy, when this knowledge is acquired, to unravel the difeafed vifcera, which, without it, muft appear confufed and intricate.

"Active

"Active inflammation should be distinguished from turgidity of the vessels; for often a fullness of the veins, mechanically produced, is described as an active inflammation in the brain and in the pleura, and still oftener in the abdomen. In dropsy, in violent distension of the intestines, in tympanites intestinalis, and after child-bearing, the veins of the intestines and peritoneum are often found distended with blood. But in real inflammation, the peritoneum becomes thickened, pulpy, and less transparent — the blood is also of a brighter red colour; a circumstance which seems not to be owing to a peculiar property in the inflamed part, of preserving the arterial colour of the blood (as Mr. Hunter suggests), but to its more general suffusion.

"As the eye becomes dry and painful and inflamed when the eye-lids are forcibly kept open and prevented from spreading the secretion upon its surface; so, when the enveloping membrane of the viscera is exposed, the natural secretion of its surface is destroyed, and it is irritated and inflamed. Or, by inflammation from any other cause, the secretion is destroyed; the parts lying in contact are no longer kept separate; they mutually affect each other; and producing a new action, unite.

"Adhesions are produced in the peritoneum and intestines in a wonderfully short time; and the smooth membrane, when it is torn from its new connections, appears cellular; or, upon being cut, thickened, and solid — or if the surface have undergone severe inflammation (without being allowed to form these adhesions which are so frequently the consequence of inflamed peritoneum), its surface becomes ragged, and numerous floculi of new membranes are formed upon it.

"In diseases where inflammation has spread among the viscera, it is generally understood that the peritoneum is the original seat of the inflammation.—And according to this view of the subject, it appears upon dissection, that the intestines do more readily than the muscles participate in the inflammation of the peritoneum. The muscles are indeed guarded in some measure by the loose cellular substance, which separates them from the peritoneum. But this does not satisfactorily account for what in the above view appears to be so great a difference between the sympathy of the intestines and that of the muscles with the peritoneum. The true explanation seems to be, that the disease or inflammation is in general communicated, not from the peritoneum to the intestines, but from the intestines to the peritoneum.—It is the disease of the intestines which produces those deadly symptoms that are said to mark inflammation of the abdominal cavity; and although there are diseases in which the peritoneum is peculiarly the seat of inflammation; yet the inflammation of the peritoneum, produced by any external cause, is dangerous only by propagating its inflammation to the intestines."

The Author's observations on the *stomach*, *duodenum*, and *liver*, are so important, that we shall give them at length in our next Number.

---

*A Letter*

*A Letter to Dr. Percival on the Prevention of infectious Fevers, and an Addrefs to the College of Phyficians at Philadelphia on the Prevention of the American Peftilence.* By JOHN HAYGARTH, M.D. &c. 8vo. pp. 188. London, 1801. Cadell and Davies.

THIS truly philofophical and philantrophic work will doubtlefs be read by every friend of humanity, and the medical profeffion. Short extracts would convey no adequate idea of its merits; and long ones are inconfiftent with our plan; we muft therefore refer our readers to the book itfelf.

---

*Inftructions relative to Self-prefervation during the Prevalence of contagious Difeafes.* By a Phyfician. 8vo. pp. 14, price 6d. London, 1801. Seely, Callow, &c.

IN the *Advertifement* prefixed to this fhort Pamphlet, the Author obferves, that "Contagious and malignant Fevers have of late prevailed, and ftill continue to prevail, to an unufual extent, in the metropolis and other parts of the kingdom. The contagion is, unknowingly, carried from place to place by numbers; who, if they were made acquainted with the neceffary precautions and directions, would ceafe to be the propagators of infection, and would be enabled to nip the evil, whether in themfelves or others, in the bud.

"This is a fubject which concerns perfons of every defcription, but more efpecially the heads of families and proprietors of fchools. It has attracted the notice, and repeatedly employed the pen, of Dr. Haygarth, and other able and philanthropic phyficians; but none of thefe gentlemen appear to us to have treated this fubject upon a plan adapted to the ufe of the better clafs of houfekeepers; —a plan which we have attempted in the following pages.

"P. S. While this pamphlet has been printing, a new performance has appeared, from the pen of Dr. Haygarth, entitled '*A Letter to Dr. Percival, on the Prevention of Infectious Fevers, read to the Literary and Philofophical Society of Bath;*' but as this Treatife, which exhibits fo fine a fpecimen of patient refearch and juft reafoning, will, it is probable, be read only by thofe who belong to the medical profeffion; it will not fuperfede the neceffity of a fet of inftructions defigned, like the prefent, for the ufe of the community at large."

In this plain and ufeful work the following fubjects are confidered, viz.

"What is meant by a contagious fever. When it occurs in a family, what precautions fhould be obferved by the nurfes and attendants; by the relatives; by vifitors. Of the proper regimen and diet, during the prevalence of contagious difeafes. Of certain reputed prefervatives. Of the management of the fick-room."

